# Mortality in the Melbourne injecting drug user cohort study (MIX)

**DOI:** 10.1186/s12954-015-0089-3

**Published:** 2015-12-09

**Authors:** Dhanya Nambiar, Paul A. Agius, Mark Stoové, Matthew Hickman, Paul Dietze

**Affiliations:** Centre for Population Health, Burnet Institute, Melbourne, Australia; Department of Epidemiology & Preventive Medicine, Monash University, Melbourne, Australia; School of Social & Community Medicine, University of Bristol, Canynge Hall, Bristol, UK

**Keywords:** Emergency services, Australia, Injecting drug use, Mortality, Cohort

## Abstract

**Background:**

There are few studies of mortality amongst people who inject drugs (PWID) in Australia. In this study, we estimate mortality in a cohort of PWID in Melbourne and examine predictors of mortality including health service use, demographic characteristics, drug use and personal wellbeing.

**Findings:**

We linked identifiers from the Melbourne injecting drug use cohort study (MIX; *n* = 655) to the National Death Index from 2008 to 2012 to estimate standardised mortality ratios (SMRs). Cox regression was used to examine the bivariate relationship between exposures determined at baseline and subsequent mortality. There were 24 (3.6 %) deaths over the study period. The mortality rate in the cohort was 1.0 per 100 PY (95 % CI 0.71–1.57), with an SMR of 17.3 (95 % CI 11.6–25.8). Baseline reports of four or more lifetime incarcerations (HR 3.65, 95 % CI 1.16–11.52), past month ambulance attendance (HR 4.43, 95 % CI 1.76–11.17), past month emergency department presentation (HR 3.44, 95 % CI 1.47–8.03) and past 6-month self-reported heroin overdose (HR 3.14, 95 % CI 1.24–7.96) were associated with increased mortality risk.

**Conclusions:**

Contact with emergency services, particularly for drug overdose, remains a lost opportunity to provide referrals for harm reduction and naloxone training programmes to PWID at greater risk of mortality.

**Electronic supplementary material:**

The online version of this article (doi:10.1186/s12954-015-0089-3) contains supplementary material, which is available to authorized users.

## Introduction

Estimates of mortality among people who inject drugs (PWID) in developed countries (based mostly on treatment samples [1]) range between 0.5 and 5 deaths per 100 person-years (PY). Stoové et al. [2] reported a mortality rate of 0.8 per 100 PY among PWID in Melbourne between 1990 and 2006, with most deaths related to drug overdose and other injecting-related causes; unlike other countries, HIV mortality was uncommon [3].

A range of factors increase mortality risk among PWID, including having a history of non-fatal overdose events [4]. PWID who overdose often come into contact with ambulance and emergency department (ED) services; however, the relationship between the use of these and other health services with mortality has not been previously explored.

We provide updated estimates of mortality among PWID in Australia where HIV prevalence is low and targeted health services for PWID are available [5]. We describe the relationship between mortality and demographic characteristics, patterns of drug use, measures of personal wellbeing and use of health services.

## Methods

### Data collection

Baseline data from the Melbourne injecting drug user cohort study (MIX) collected in Melbourne between November 2008 and March 2010 was linked to Australia’s National Death Index (NDI). MIX participants were recruited through a combination of respondent-driven sampling, snowball sampling and outreach. Eligible participants were aged 18 and over, reported injecting regularly for at least 6 months and had a valid Medicare card. The baseline MIX questionnaire included questions on socio-demographics, drug use, health service use, and self-reported health and wellbeing. Further details of the recruitment methods and the wider study are described elsewhere [6]. Probabilistic data linkage of participant identifier codes based on letters from first and last names, gender and date of birth was used to link NDI data, with matches validated using full first names, deaths known to the study team and date of last contact. Deaths up to December 2012 were captured across all states and territories. Cause-of-death codes are reported where available.

The study was approved by the Human Research Ethics Committee of the Victorian Department of Health and the Monash University Human Research Ethics Committee, and informed consent was obtained from all participants.

### Statistical analyses

Person-years (PY) were calculated as the time from baseline interview until death or censorship at 31st December 2012. Mortality rates were calculated using the person-time method [7] and disaggregated by gender. Standardised mortality ratios (SMRs) compared observed age- and sex-specific mortality rates of the cohort with age- and calendar-year-matched Australian population estimates using indirect standardisation. Five-year age groups were assigned. The 95 % confidence intervals (CI) were calculated using the Poisson distribution.

Semi-parametric analysis using Cox regression was used to describe baseline characteristics associated with mortality, including socio-demographic, drug use and health service use data. Validated tools included a variant of the AUDIT-C scale [8] to assess past month alcohol consumption (categorised as abstinent, 1–7, 8+) and the SF8 quality of life scale summary measurements for physical and mental health [9] as measures of health-related quality of life (categorised as low (lower quartile scores), average (second and third quartile scores) and high (upper quartile scores) [10]). The small number of events (deaths) restricted analyses to bivariate relationships between exposures and mortality. Variables were considered significant at *p* < 0.05. Associations were reported using hazard ratios (HR); 95 % confidence intervals (CIs) for crude mortality rates were calculated using a Poisson distribution. We describe time-varying factors associated with mortality using longitudinal data from the MIX cohort as supplementary analyses to identify missed opportunities for interventions [2, 7]. Analyses were conducted using Stata Version 13.1 (StataCorp LP, TX, USA).

## Results

### Baseline characteristics

We recruited 688 participants between 2008 and 2010, of whom 23 (3.4 %) were excluded from analysis due to incomplete data on exposures of interest such as past month use of emergency, outpatient and ambulance services. No mortality cases were excluded in this way. Participants with complete data (*n* = 665) contributed 2277 PYs across a median of three interviews. Table 1 describes baseline characteristics of participants who were young relative to other studies of Australian PWID (median age 29, interquartile range (IQR) 17–40), predominantly male and unemployed. Participants had been injecting for a median of 10 years (IQR 6–13) and over a third were receiving OST. Participants were frequent users of health services and almost one-third had poor self-reported physical health.

**Table 1:** Baseline socio-demographic, risk and health service utilisation factors associated with mortality among PWID in Melbourne (*n* = 665)

Characteristics	Baseline *n* (%)	Deaths *N* (%)	Hazard ratio (95 % CI)	*P* value
Gender				
Male	446 (67.1)	18 (4.0)	1.45 (0.58–3.66)	0.427
Female	219 (32.9)	6 (2.7)	1	
Employed				
Yes	91 (13.7)	2 (2.2)	0.57 (0.13–2.41)	0.444
No	574 (86.3)	22 (3.8)	1	
Stable accommodation				
Yes	539 (81.0)	22 (4.1)	2.53 (0.59–10.77)	0.209
No	126 (18.9)	2 (1.6)	1	
Current OST				
Yes	236 (35.5)	8 (3.4)	0.92 (0.39–2.16)	0.854
No	429 (64.5)	16 (3.7)	1	
Attended emergency department in the past month				
Yes	88 (13.2)	6 (12.0)	3.44 (1.47–8.03)*	0.004
No	577 (86.8)	18 (2.9)	1	
Attended PWID specific primary care services in the past month				
Yes	116 (17.4)	22 (4.0)	0.43 (0.10–1.82)	0.249
No	549 (82.6)	2 (1.7)	1	
Attended GP services in the past month				
Yes	387 (58.2)	16 (4.1)	1.44 (0.61–3.36)	0.403
No	278 (41.8)	8 (2.9)	1	
Attended ambulance services in the past month				
Yes	50 (7.5)	6 (12.0)	4.43 (1.76–11.17)*	0.002
No	615 (92.5)	18 (2.9)	1	
Past week heroin injecting frequency				
None	10 (25.6)	6 (3.5)	1	
Less than daily	297 (44.7)	12 (4.0)	1.14 (0.43–3.04)	0.794
Daily or more	198 (29.8)	6 (3.0)	0.84 (0.27–2.60)	0.760
AUDIT Alcohol Consumption Questions score				
Abstinent	239 (35.9)	9 (3.8)	1	
1–7	264 (39.7)	7 (2.6)	0.69 (0.26–1.87)	0.472
8 +	162 (24.4)	8 (4.9)	1.35 (0.52–3.50)	0.537
Arrested in the past 12 months				
Yes	359 (54.0)	17 (4.7)	2.11 (0.88–5.09)^†^	0.096
No	306 (46.0)	7 (2.3)	1	
Frequency of past incarceration				
None	271 (40.7)	7 (2.6)	1	
3 or less	339 (51.0)	12 (3.5)	1.39 (0.55–3.52)	0.491
4 or more	55 (8.3)	5 (9.1)	3.65 (1.16–11.52)*	0.027
Duration of injecting career (years)				
Less than 3	77 (11.6)	4 (5.2)	1	
3–9	243 (36.5)	8 (3.3)	0.63 (0.19–2.09)	0.450
Greater than 10	345 (51.9)	12 (3.5)	0.67 (0.22–2.08)	0.491
Overdose in the past 6 months				
Yes	10 (1.5)	6 (9.2)	3.14 (1.24–7.96)*	0.016
No	655 (98.5)	18 (3.0)	1	
SF8 physical health score in the past month				
High (>56)	161 (24.1)	4 (2.5)	1	
Average (45–56)	330 (49.6)	10 (3.0)	1.23 (0.39–3.93)	0.725
Low (<45)	174 (26.2)	10 (5.7)	2.39 (0.75–7.63)^†^	0.140
SF8 mental health score in the past month				
High (>53)	181 (27.2)	8 (4.4)	1	
Average (31–53)	327 (49.2)	9 (2.7)	0.63 (0.24–1.64)	0.344
Low (<31)	157 (23.6)	7 (4.5)	1.03 (0.37–2.85)	0.949

*p < 0.05

^†^ *p* < 0.1

### Mortality rates, causes of death, and correlates of mortality

There were 24 (3.6 %) deaths in study period; the median age at death was 28 years (range 19–31) and the median time from the last interview to death was 257 days (range 7–1134 days). The estimated mortality rate was 1.0 per 100 PY (95 % CI 0.7–1.6). The estimated SMR was 17.3 (95 % CI 11.6–25.8) overall and there was no evidence of a significant difference between women (23.5, 95 % CI 10.5–52.3) and men (15.9, 95 % CI 10.0–25.3).

Cause of death was available for 23 of the 24: 12 were drug-related, involving opioids and multiple drug use, four were suicides, four were due to physical assault, two were from medical conditions not directly related to injecting drug use, and one resulted from an accident-related injury. Heroin was the most common drug detected in the drug-related deaths (83 %), followed by benzodiazepines (67 %). Most (83 %) drug-related deaths involved multiple substances, including heroin, benzodiazepines, methadone, antidepressants, antipsychotics and alcohol.

Bivariate Cox regression analyses of associations between mortality and exposures are presented in Table 1. Increased mortality risk was associated with frequency of past incarceration (four or more compared to none), reporting past month ambulance attendance, reporting past month ED presentation and reporting a heroin overdose in the past 6 months. The supplementary analyses of time-varying exposures showed the same pattern, but some exposures failed to reach significance (Additional file 1: Table S1).

## Discussion

The excess mortality reported in this study was almost three times higher than previous Australian estimates involving PWID and people on opioid substitution therapy (OST) [2, 11]. The excess mortality was also higher than reported in other countries with a low HIV prevalence [12]. The heightened risk of mortality in this community-based cohort might be due to systematic differences beyond injecting drug use. The cohort was community-recruited rather than treatment-recruited [11] and was relatively young compared to other cohorts [2, 11]. The excess mortality is comparable to results in contemporaneous cohorts of similar age [7], and the mortality rate might converge to estimates in the wider literature over a longer observation period potentially reflecting changes in risk behaviour with age and retention in drug treatment observed in other studies [11, 13].

The mortality rate reported here is comparable to that in a recent Australian cohort of PWID recruited from prison settings and followed post-release [14]. Over half the MIX cohort reported a history of incarceration at baseline and we found that frequent incarceration was associated with mortality. Multiple events of incarceration indicate a particularly vulnerable group with an over-representation of low levels of education and socioeconomic status, and unsafe injecting practices [15]. The primary overdose prevention tools potentially available through the criminal justice system are the pre- to post-release continuation of OST and the provision of naloxone at prison release. The impact of such programmes can be enhanced through a continuum of care between prison and community health services that facilitates programme retention.

Ambulance and ED attendance reported in the month prior to the last interview were associated with increased risk of mortality, as was reporting a heroin overdose in the previous 6 months. For three of the five deaths for which a previous ambulance attendance was reported, the attendance related to an overdose (data not shown). Frequent contact with emergency and ambulance services could be used to identify PWID at greater risk of fatal overdoses. Multiple overdose events may indicate reduced tolerance, interactions with other substances and the presence of other comorbidities [16]. Contact with emergency services is an opportunity to provide targeted harm reduction initiatives, such as take-home naloxone.

### Limitations

Our study was limited by a small number of mortality events which affected the precision of estimates and limited adjusted mortality models. In most cases there was only a short observation period available for participants who died which limited our ability to observe the effect of time-varying risk factors, although this strengthens the temporal relevance of the observations in relation to mortality. The quality of the linkage was dependent on the accuracy of participant-provided identifier data. Exposures in cohort studies such as this are assumed to be fixed between interviews or censorship; however, this assumption is not always valid and means the predictors of mortality reported here should be interpreted with caution.

## Conclusions

This analysis of mortality in community-recruited Australian PWID found significantly higher excess mortality than previous Australian studies and studies in comparable developed countries. These results highlight the importance of tailored interventions prioritising overdose prevention to young PWID. Our findings point to prison and emergency settings as ideal opportunities to deliver interventions such as harm reduction messages and referrals for naloxone training and prescribing to reduce mortality [17].

### Highlights

- We describe mortality in a cohort of young, community-based Australian PWID
- Excess mortality in the cohort was higher than other local estimates and settings where HIV prevalence is low
- Recent use of emergency services such as emergency departments and ambulances was associated with mortality
- Opportunities remain in prisons and emergency settings to deliver overdose-related interventions

## Additional file

Additional file 1: Table S1. Time-varying socio-demographic, risk and health service utilisation factors associated with mortality among PWID in Melbourne (*n* = 665). (DOC 99 kb)
