# Supplementary material for: Mortality in the Melbourne injecting drug user cohort study (MIX)
Source: Harm Reduct J. 2015 Dec 9;12:55. doi: 10.1186/s12954-015-0089-3 (PMC4674911; doi:10.1186/s12954-015-0089-3)
Supplement: Additional file 1: Table S1. — Time-varying socio-demographic, risk and health service utilisation factors associated with mortality among PWID in Melbourne (n = 665). (DOC 99 kb) [file 12954_2015_89_MOESM1_ESM.doc]

Additional file 1: Table S1: Time-varying socio-demographic, risk and health service utilisation factors associated with mortality among PWID in Melbourne (n=665).

| Characteristics | Baseline  n (%) | Hazard Ratio (HR) | P-value |
| --- | --- | --- | --- |
| **Gender** |  |  |  |
| **Male** | 446 (67.1) | 1.45 (0.58 – 3.66) | 0.427 |
| **Female** | 219 (32.9) | 1 |  |
| **Employed** |  |  |  |
| **Yes** | 91 (13.7) | 0.66 (0.20 - 2.22) | 0.518 |
| **No** | 574 (86.3) | 1 |  |
| **Stable accommodation** |  |  |  |
| **Yes** | 539 (81.0) | 1.02 (0.35 – 2.98) | 0.986 |
| **No** | 126 (18.9) | 1 |  |
| **Current OST** |  |  |  |
| **Yes** | 236 (35.5) | 0.80 (0.35 - 1.84) | 0.591 |
| **No** | 429 (64.5) | 1 |  |
| **Attended emergency department in the past month** |  |  |  |
| **Yes** | 88 (13.2) | 1.95 (0.73 - 5.23) | 0.185 |
| **No** | 577 (86.8) | 1 |  |
| **Attended PWID specific primary care services in the past month** |  |  |  |
| **Yes** | 116 (17.4) | 1.97 (0.82 – 4.75) | 0.132 |
| **No** | 549 (82.6) | 1 |  |
| **Attended GP services in the past month** |  |  |  |
| **Yes** | 387 (58.2) | 1.18 (0.50 - 2. 77) | 0.705 |
| **No** | 278 (41.8) | 1 |  |
| **Attended ambulance services in the past month** |  |  |  |
| **Yes** | 50 (7.5) | 3.56 (1.33 – 9.53)* | 0.011 |
| **No** | 615 (92.5) | 1 |  |
| **Past week heroin injecting frequency** |  |  |  |
| **None** | 10 (25.6) | 1 |  |
| **Less than daily** | 297 (44.7) | 1.37 (0.53 – 3.57) | 0.521 |
| **Daily or more** | 198 (29.8) | 1.19 (0.40 – 3.56) | 0.767 |
| **AUDIT Alcohol Consumption Questions score** |  |  |  |
| **abstinent** | 239 (35.9) | 1 |  |
| **1 – 7** | 264 (39.7) | 0.86 (0.30 – 2.47) | 0.804 |
| **8 +** | 162 (24.4) | 2.21 (0.84 – 5.80) | 0.110 |
| **Arrested in the past 12 months** |  |  |  |
| **Yes** | 359 (54.0) | 2.21 (0.91 – 5.32) † | 0.915 |
| **No** | 306 (46.0) | 1 |  |
| **Frequency of past incarceration** |  |  |  |
| **None** | 271 (40.7) | 1 |  |
| **3 or less** | 339 (51.0) | 2.71 (0.77 – 9.47) | 0.158 |
| **4 or more** | 55 (8.3) | 5.01 (1.27 – 19.78)* | 0.011 |
| **Duration of injecting career (years)** |  |  |  |
| **less than 3** | 77 (11.6) | 1 |  |
| **3–9** | 243 (36.5) | 0.58 (0.15 – 2.22) | 0.436 |
| **greater than 10** | 345 (51.9) | 0.56 (0.16 – 1.99) | 0.374 |
| **Overdose in the past 6 months** |  |  |  |
| **Yes** | 10 (1.5) | 2.59 (0.97 – 6.95) † | 0.059 |
| **No** | 655 (98.5) | 1 |  |
| **SF8 physical health score in the past month** |  |  |  |
| **High (>56)** | 161 (24.1) | 1 |  |
| **Average (45–56)** | 330 (49.6) | 1.34 (0.42 – 4.29) | 0.618 |
| **Low (< 45)** | 174 (26.2) | 2.69 (0.84 - 8.60) † | 0.094 |
| **SF8 mental health score in the past month** |  |  |  |
| **High (>53)** | 181 (27.2) | 1 |  |
| **Average (31–53)** | 327 (49.2) | 0.45 (0.17 – 1.26) | 0.129 |
| **Low (<31)** | 157 (23.6) | 1.31 (0.51 – 3.41) | 0.572 |

*p<0.05

†p<0.1
